# Ku80 cooperates with CBP to promote COX-2 expression and tumor growth

**DOI:** 10.18632/oncotarget.3508

**Published:** 2015-03-10

**Authors:** Yao Xiao, Jingshu Wang, Yu Qin, Yang Xuan, Yunlu Jia, Wenxian Hu, Wendan Yu, Meng Dai, Zhenglin Li, Canhui Yi, Shilei Zhao, Mei Li, Sha Du, Wei Cheng, Xiangsheng Xiao, Yiming Chen, Taihua Wu, Songshu Meng, Yuhui Yuan, Quentin Liu, Wenlin Huang, Wei Guo, Shusen Wang, Wuguo Deng

**Affiliations:** ^1^ Institute of Cancer Stem Cell & First Affiliated Hospital, Dalian Medical University, Dalian, China; ^2^ Sun Yat-sen University Cancer Center, State Key Laboratory of Oncology in South China, Collaborative Innovation Center of Cancer Medicine, Guangzhou, China; ^3^ Department of Surgical Oncology, Sir Run Run Shaw Hospital, Zhejiang University, Hangzhou, China; ^4^ State Key Laboratory of Targeted Drug for Tumors of Guangdong Province, Guangzhou Double Bioproduct Inc., Guangzhou, China

**Keywords:** Ku80, CBP, COX-2, lung cancer

## Abstract

Cyclooxygenase-2 (COX-2) plays an important role in lung cancer development and progression. Using streptavidin-agarose pulldown and proteomics assay, we identified and validated Ku80, a dimer of Ku participating in the repair of broken DNA double strands, as a new binding protein of the COX-2 gene promoter. Overexpression of Ku80 up-regulated COX-2 promoter activation and COX-2 expression in lung cancer cells. Silencing of Ku80 by siRNA down-regulated COX-2 expression and inhibited tumor cell growth *in vitro* and in a xenograft mouse model. Ku80 knockdown suppressed phosphorylation of ERK, resulting in an inactivation of the MAPK pathway. Moreover, CBP, a transcription co-activator, interacted with and acetylated Ku80 to co-regulate the activation of COX-2 promoter. Overexpression of CBP increased Ku80 acetylation, thereby promoting COX-2 expression and cell growth. Suppression of CBP by a CBP-specific inhibitor or siRNA inhibited COX-2 expression as well as tumor cell growth. Tissue microarray immunohistochemical analysis of lung adenocarcinomas revealed a strong positive correlation between levels of Ku80 and COX-2 and clinicopathologic variables. Overexpression of Ku80 was associated with poor prognosis in patients with lung cancers. We conclude that Ku80 promotes COX-2 expression and tumor growth and is a potential therapeutic target in lung cancer.

## INTRODUCTION

Lung cancer is the leading cause of cancer-related death in the world [1]. Although the advances in diagnosis and treatment have been achieved, the five-year survival rate of lung cancer remains very low. Cancer metastases during lung carcinoma development is the major cause of high mortality and low survival rate [2]. Furthermore, the complexity and the real-time change of the key biomarkers during the process of lung carcinogenesis also caused its therapeutic difficulties [3-6]. Therefore, discovering and understanding the regulatory mechanisms of lung carcinogenesis has become increasingly important to provide potentially effective therapeutic targets.

Cyclooxygenase-2 (COX-2), an important inflammation factor in cancer development and progression, has been extensively studied [7, 8]. As an inducible isoform of COX, COX-2 can convert archidonic acid into prostaglandin H2. It is inducible in response to certain stimuli such as growth factors and cytokines and is involved in many pathological processes such as inflammation and carcinogenesis [9, 10]. It was reported that more than 15% of malignant tumors were correlated with infection [11]. It has been well established that COX-2 is up-regulated in a variety of cancers and promotes tumor growth [12, 13]. Overexpression of COX-2 and its metabolite prostaglandin E2 (PGE2) have been reported to contribute to increased angiogenesis [14, 15], apoptosis resistance [16-18], decreased host immunity [19, 20], and enhanced invasion and metastasis [21, 22].

The COX-2 gene, located at chromosome 1q^25.2-25.3^, is composed of 10 exons and 9 introns with approximately 8.3 kb in size [23]. There are CAAT/enhancer binding protein (C/EBP) and cAMP response elements in the 5′-terminal nucleotide sequence. There are also some other protein binding sites in these gene sequences, such as the activator protein-2 (AP-2), the nuclear factor-kappa B (NF-κB) [24, 25] and some transcriptional co-activators such as p300/CBP [26,27]. The expression of COX-2 is strictly and specifically regulated by these proteins. However, it remains unclear about how COX-2 is over-activated during carcinogenesis. We postulated that there were some unknown tumor-specific COX-2 promoter-binding proteins to regulate COX-2 expression in human cancers. In this study, we use streptavidin-agarose pulldown assay and proteomics [28] techniques to discover and identify these potentially critical unknown regulatory factors of COX-2 in human lung cancer cells. One of the proteins was identified as Ku80, a DNA repair protein. It was detected in the COX-2 promoter DNA-protein complexes eluted from nuclear extracts prepared only from lung cancer cells.

Ku80 is a dimer of Ku, which is the regulatory DNA-binding region of the DNA-dependent protein kinase (DNA-PK). Ku80 has been implicated in several nuclear processes, including the repair of broken DNA double strands and V(D)J recombination [29], telomere maintenance, antigen receptor gene arrangements, regulation of specific gene transcription, apoptosis, regulation of heat shock-induced responses, as well as a newly identified role in regulation of the G2 and M phases of the cell cycle [30, 31]. Ku protein itself has also been reported to be able to function as transcription factors and bind in a sequence-speciﬁc manner to promoter elements [32]. For example, Ku86 binds to the promoter of the heat shock proteins, glucose-regulated peptide78, grp94 [33] and S100A9 and regulates their gene expression [34]. Interestingly, Ku70/80 also mediates adhesion of cells to fibronectin, which indicates its role as an adhesion receptor. Ku80 also appears to be coupled with signal transduction [35]. Recent reports suggest that there is a positive relationship between Ku and the development of cancer, making Ku an important candidate target for anticancer drug development [36,37]. Specifically, prior studies suggest that a delicate balance exists in Ku expression, as overexpression of Ku proteins promotes oncogenic phenotypes, including hyper-proliferation and resistance to apoptosis; whereas deficient or low expression of Ku leads to genomic instability and tumorigenesis [38, 39]. Inhibition of the expression of either Ku70 or Ku80 results in the inhibition of cell growth, the induction of apoptosis [40, 41] and attenuation of nuclear NF-κB p50 activity [42]. On the contrary, overexpression of Ku protein increases nuclear NF-κB activity in Rat ﬁbroblast [43]. However, the role of Ku80 or Ku70 in regulating cancer-related gene expression remains unclear enough, especially whether Ku80 or Ku70 controls COX-2 expression and activity directly or indirectly in lung carcinoma cells is largely unknown.

In this study, we used biotin-streptavidin pulldown assay to look for and identify the unknown and essential transcriptional regulators for COX-2 and found that Ku80 specifically bound to the COX-2 promoter and activated COX-2 transcription, and then increased COX-2 expression and the production of its downstream molecule PGE_2._ We also studied the clinical association between Ku80 and COX-2 in lung cancer tissue samples. In addition, we also investigated the possible association between CBP and Ku80 in mediating COX-2 transcription. All the results support our initial discovery that Ku80 may act as an important regulator of COX-2 expression to participate in the development of lung cancer. Based on the important role of COX-2 in inflammation and infection-associated tumorigenesis, our findings, to some extent, suggests that Ku80 may serve as a potential novel therapeutic target for human lung cancer.

## RESULTS

### Discovery of COX-2 promoter-binding proteins in lung cancer cells

**Figure 1:**
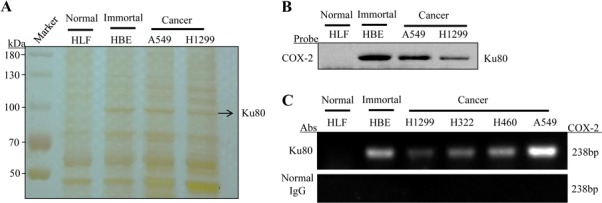
Ku80 was identified and validated as specific binding protein of COX-2 promoter in lung cancer cells (A). The streptavidin-biotin pulldown assay was performed to find out the specific proteins which bind to COX-2 promoter. Nuclear extracts prepared from human lung cancer cell lines (H1299 and A549), immortalized lung cell line (HBE) and normal lung cell lines (HLF) were incubated with biotin-labeled COX-2 promoter probe (−508 to +30) and streptavidin-agarose beads. The DNA-protein complexes were separated by SDS-PAGE, and protein bands were visualized by silver staining. The arrow indicates the candidate COX-2 promoter-binding protein. (B). Immunoblot assay for detection of Ku80 binding to COX-2 promoter probe (−508 to +30). Ku80 protein in the DNA-protein complexes was detected by Western blot assay using anti-Ku80 antibody. (C). Chromatin immunoprecipitation assays were carried out using the COX-2 promoter from normal lung cell, immortal lung cell and various lung adenocarcinoma cells. PCR products were separated on 2% agarose gels. Normal IgG was negative control of Ku80 antibody.

According to our previous study [44], we designed and synthesized a 479-bp biotin-labeled double-stranded oligonucleotide probe corresponding to the 5′-flanking sequence of the COX-2 gene from −30 to −508 as a DNA probe to pull down the transcriptional factors of COX-2 gene and to assess their binding on COX-2 promoter region. Nuclear extracts prepared from human lung cancer cell lines (H1299 and A549), immortalized lung cell line (HBE) and normal lung cell lines (HLF) were incubated with biotin-labeled COX-2 promoter probe and streptavidin-agarose beads. After washing and elution, the COX-2 promoter-binding proteins were separated by SDS-PAGE and visualized through silver staining. As shown in Figure 1A, one of the protein bands (at about 90-100 kDa) significantly appeared in the lung cancer cells and immortalized cells with high COX-2 expression but not in HLF cells. Next, we used proteomics approach to identify this specific COX-2 promoter-binding protein. The protein band was dissected from the gel and digested with trypsin. The peptide digests were further analyzed by mass spectrum and identified by searching an internationally recognized proteomics data library. The protein band was predicted to be Ku80, also called as X-ray repair complementing defective repair in Chinese hamster cells 5 (XRCC5).

### Validation of Ku80 as a COX-2 promoter-binding protein

To verify the binding of Ku80 at COX-2 promoter region in lung cancer cells, we performed immunoblot analyses for the proteins eluted from the biotin-streptavidin pulldown complexes using anti-Ku80 antibody. As shown in Figure 1B, the Ku80 was clearly detected in the complex prepared from the human lung cancer cell lines H1299, A549 and immortal cell line HBE but not from the normal lung cell line HLF, demonstrating the tumor cell-selective binding of Ku80 to the COX-2 promoter. To further confirm that Ku80 functions as a special COX-2 promoter-binding protein *in vivo*, we analyzed the binding of Ku80 to the chromatin COX-2 promoter in living cells by ChIP assay using a specific antibody against Ku80. Normal IgG was used as negative control. As shown in Figure 1C, COX-2 promoter was amplified in lung cancer and immortalized cells but not in normal lung cells, indicating the binding of Ku80 protein at the endogenous COX-2 promoter again. Compared to the three lung cancer cell lines A549, H460 and H322 and immortalized cell HBE with high expression of COX-2, a relatively weak Ku80 binding on COX-2 promoter was detected in normal lung cell line HLF and lung cancer cell H1299 with low expression of COX-2. The DNA binding was undetectable when using a normal IgG control in the ChIP assay. These results confirmed that Ku80 specifically bound to the COX-2 promoter in human lung cancer cells.

### Regulation of Ku80 on COX-2 transcription activity and expression

**Figure 2:**
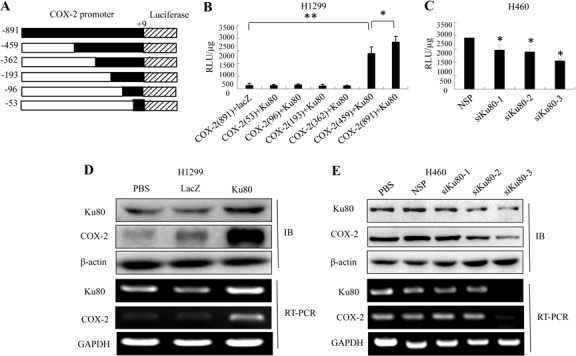
Ku80 bound to the COX-2 promoter region and regulated its transcriptional activation in lung cancer cells (A). A 5′-flanking DNA fragment from position −891 to +9 (−891/+9, −459/+9, −363/+9, −193/+9, −96/+9, −53/+9) of human COX-2 gene was constructed into a promoter-driven luciferase expression vector, pGL3. (B). H1299 cells were cotransfected with Ku80 and different COX-2 promoter-driven luciferase plasmids for 48 hrs. The proteins were extracted, and luciferase activity was detected by luciferase reporter assay kit. FLAG-lacZ plasmids were negative control. (C). H460 cells were cotransfected with siKu80 and COX-2 promoter-driven luciferase (−459/+9) plasmids. Luciferase activity was detected as described before. (D). Up-regulation of COX-2 mRNA and protein expression by Ku80 overexpression. H1299 cells were transfected with 2 μg/mL of Ku80 plasmid or negative control lacZ for 48 h, and the expression of COX-2 mRNA and protein were analyzed by RT-RCR and Western blot. (E). Down-regulation of COX-2 mRNA and protein expression by Ku80 knockdown. H460 cells were transfected with 1 μg/mL of Ku80siRNA or nonspeciﬁc control siRNA for 48 h, and the expression of COX-2 mRNA and protein were analyzed by RT-PCR and Western blot

Based on the results described above, we hypothesized that Ku80 might drive the transcription of COX-2 as a transcription regulator in lung cancer cells. To verify this, we constructed luciferase-reporter vector driven by COX-2 promoter with six different lengths (shown in Figure 2A). We co-transfected H1299 cells, which had low Ku80 expression, with the plasmids expressing Ku80 and a luciferase reporter driven by COX-2 promoter. As shown in Figure 2B, the luciferase expression was higher in cells co-transfected with Ku80 and COX-2 (−459/+9 and −891/+9)-luciferase plasmids compared with those in cells co-transfected with Ku80 and COX-2-luciferase plasmids with other lengths of COX-2 promoter regions, or cells co-transfected with LacZ and COX-2-luciferase plasmids. This result not only demonstrated that Ku80 drove the transcription of COX-2, but also indicated that Ku80 bound to the region of COX-2 promoter at −363 to −459.

To further verify the role of Ku80 in regulating COX-2 transcription, we blocked the expression of endogenous Ku80 using its specific siRNA in human lung cancer cells H460. We co-transfected H460, which had high expression of Ku80, with a Ku80-specific siRNA and a COX-2 (−459/+9)-luciferase plasmid. At 48 hours after treatment, inhibition of Ku80 expression by siKu80-1, siKu80-2 or siKu80-3 attenuated the activity of COX-2 promoter (−459/+9) compared with negative control group (Figure 2C). The results indicated again the role of Ku80 as a transcriptional factor to drive the transcription of COX-2 in human lung cancer cells. Next, we further evaluated the effect of Ku80 on COX-2 expression at protein and mRNA levels. We found that the ectopic expression of Ku80 significantly increased COX-2 expression at the levels of protein and mRNA in H1299 cells (Figure 2D). By contrast, the knockdown of Ku80 by Ku80 siRNA significantly decreased COX-2 expression in H460 cells (Figure 2E).

### Regulation of Ku80 on lung cancer cell proliferation and migration

**Figure 3:**
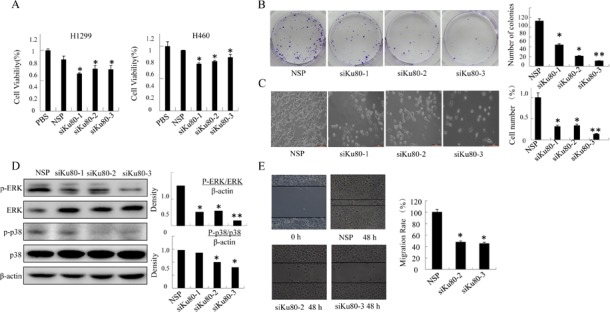
Ku80 regulated the growth of lung cancer cells by inhibiting MAPK pathways (A). Cell viability was analyzed by MTT in H1299 and H460 cells transfected with 1 μg/mL siKu80 or nonspecific control siRNA for 48h were observed. (B). Colony formation assay of H460 cells transfected with 1 μg/mL siKu80 or nonspecific control siRNA twice a week for two weeks was performed. The quantification of colonies was shown. (C). The morphology of H460 cells transfected with 1 μg/mL siKu80 or nonspecific control siRNA for 48h were observed. The cell number was shown. (D). MAPK, p38 and their phosphorylation in H460 cell transfected with siKu80 was respectively detected by Western blot assay using antibodies against p-ERK, ERK, p-p38, p38. And the quantitative measurement was shown below the Western blot. (E). Cell migration was analyzed by a wound-healing assay. H460 cells were seeded in 6-well plates and grown to full confluence. Cell migration was measured as described before, and the migration rate was calculated. The data are presented as the mean ± SD of three separate experiments. (*P<0.05, **P<0.01).

Considering that the role of COX-2 in promoting cancer cell growth has been well established [9] and the results above indicated its regulation by Ku80, we speculate Ku80 could also accelerate lung cancer cell proliferation and migration. As shown in Figure 3A and 3C, inhibition of Ku80 expression with siKu80 dramatically suppressed cell proliferation in lung cancer cell lines H1299 and H460. Similarly, H460 cells transfected with siKu80 showed lower activity of colony formation (Figure 3B). In addition, Ku80 knockdown also significantly inhibited cell migration (Figure 3E).

It is well known that MAPK is the downstream of COX-2. The activation of extracellular signal-regulated protein kinases (ERK1/2) is mainly associated with cell survival, proliferation, migration and cell growth, while p38 MAPK cascades are associated with the promotion of inflammation and programmed cell death [45,46]. We detected the MAPK family protein expression, and verified the role of Ku80 in activating MAPK proteins. As is shown in Figure 3D, the phosphorylation of ERK and p38 was inhibited by Ku80 knockdown, but the total level was not changed. From the results above, we concluded that Ku80 is specifically expressed in lung cancer cells and correlated with cell growth and proliferation partially through regulating MAPK signaling pathway.

### Overexpression of Ku80 and COX-2 in lung carcinoma cells and tissues and their association with patients' survival

**Figure 4:**
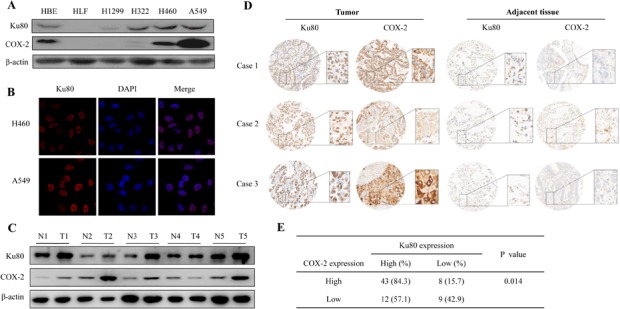
Ku80 and COX-2 were highly expressed in lung carcinoma cell and lung carcinoma tissues (A). Ku80 and COX-2 expression in lung cancer cells, HLF and HBE cell lines were detected by Western blot using anti-Ku80 and COX-2 antibody. (B). The expression of Ku80 in lung cancer cells (H460 and A549) was detected by Immunofluorescence assay. The localization of Ku80 was shown. The red staining by secondary tetra methyl rhodamine isothiocyanate-conjugated antibodies was Ku80, and the blue staining by DAPI was nuclear. (C). The protein samples extracted from five couple of human lung carcinoma tissues and adjacent tissues were used to detected the expression of Ku80 and COX-2. (D) The immunostaining analysis of Ku80 and COX-2 protein expression from human lung adenocarcinoma tissue microarray. High expression of Ku80 and COX-2 were shown by being stained as brown, and low or no expression was shown in light. Case 1, Case 2 and Case 3 mean three representative cases respectively from three different patients. Tumor and adjacent tissues were from one patient for each case. (E). The correlation between the expression of Ku80 and COX-2 in lung carcinoma tissues from 72 patients was shown.

To confirm the correlation of Ku80 and COX-2 and their biological and clinicopathologic significance in lung cancer patients, we first detected the expression of Ku80 and COX-2 in lung cancer cells and normal lung cells by Western blot. As shown in Figure 4A, Ku80 was highly expressed in lung cancer cell line H322, A549, H460 and immortalized cell line HBE, whereas its expression was lower in lung cancer cell line H1299 and normal lung cell HLF. While COX-2 nearly showed the same expression trend with Ku80 in these cell lines. Next, immunofluorescence analysis was used to further analyze the expression and sub-cellular localization of Ku80 in lung cancer cell. We found that Ku80 was highly expressed at nucleus in lung cancer H460 and A549 cells, but not in normal lung cell HLF (Figure 4B). We further detected Ku80 and COX-2 expression in lung cancer tissues and their corresponding adjacent non-cancer tissues by Western blot. Both Ku80 and COX-2 were highly expressed in lung cancer tissues compared with their adjacent non-cancer tissues (Figure 4C). Similarly, immunohistochemical staining showed that lung tumor tissues, but not their adjacent non-cancer tissues, had high expression of both Ku80 and COX-2 (Figure 4D). Three representative cases (Case 1, 2 and 3) were respectively from three different patients (Figure 4D). Among 72 patients' tumor tissue samples tested, about 43 (84.3%) patients showed high expression of both COX-2 and Ku80 (Figure 4E).

**Figure 5:**
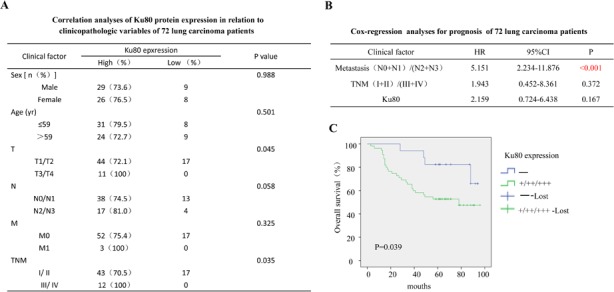
The clinical correlation analyses of Ku80 protein expression in 72 lung carcinoma patients (A). Correlation analyses of Ku80 protein expression in relation to clinicopathologic variables of 72 lung carcinoma patients. (B). Cox-regression analyses for prognosis of 72 lung carcinoma patients. (C). Kaplan-Meier analysis of overall survival with high or low Ku80 expression (P< 0.001, log-rank test).

Additionally, the correlation of Ku80 and clinicopathologic variables and prognosis of 72 lung carcinoma patients were shown in Figure 5A and B. The overall survival analysis indicated patients with low COX-2 and ku80 expression owned significantly higher survival rate compared to the patients with both high expression of these two proteins (P=0.01, Figure 5C). All the results demonstrated a potential correlation between Ku80 and COX-2 expression and their indication for the poor prognosis in lung cancer patients.

### Inhibition of tumor growth by Ku80 knockdown through down-regulating COX-2 expression in mice

**Figure 6:**
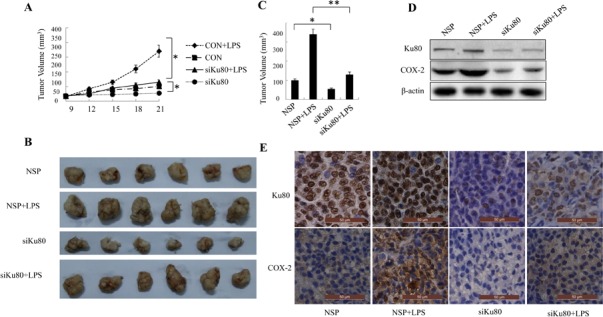
Ku80 knockdown inhibited tumor growth through down-regulating COX-2 expression in a mouse model (A). A representative picture of nude mice comparing the sizes of tumor grafts 21 days after intratumoral injection of non-specific control siRNA (CON) or Ku80-specific siRNA. (B). The morphology of tumor xenografts of each nude mice after anatomy at 21 days of treatment. (C). Tumor volume of each group of nude mice was measured and calculated as V=(width^2^×length)/2. n=6; *, P<0.05. (D). The proteins were extracted from tumor xenografts. The Ku80 and COX-2 expression was detected by Western blot. (G) Immunohistochemistry assay of Ku80 and COX-2 expression from tumor xenografts in each group of nude mice.

The effect of Ku80 on tumor growth was further examined in nude mice with human lung tumor xenografts. Human lung cancer cells H460 was injected subcutaneously into the armpit of nude mice. When the tumors were ~100 mm^3^ in size, the animals were treated with Ku80-specific siRNA and nonspecific control siRNA encapsulated by DC nanoparticles respectively. As shown in Figure 6A-C, the tumor volume and weight of mice treated with Ku80-specific siRNA was smaller and lighter compared with the group treated with non-specific RNA. To examine whether COX-2 expression is involved in Ku80-specific siRNA-mediated tumor growth suppression, we used LPS (lipopolysaccharides) to rescue COX-2 expression in mice treated with Ku80-specific siRNA, and found that LPS partially rescued the tumor growth inhibition caused by Ku80 siRNA treatment (Figure 6A-C). Furthermore, we examined the effect of Ku80 knockdown on the expression of COX-2 at protein level in xenografts by Western blot and immunohistochemistry analysis. As shown in Figure 6D-E, Ku80-specific siRNA treatment attenuated COX-2 expression in xenografts. The results above demonstrated the important role of Ku80 involved in lung tumor growth partially through regulating COX-2 expression.

### Cooperation of Ku80 with CBP to co-regulate COX-2 expression

**Figure 7:**
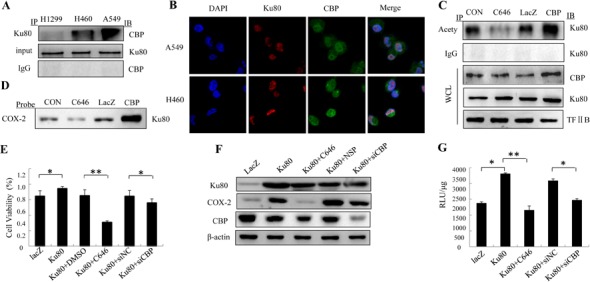
Ku80 interacted with CBP to co-regulate COX-2 expression in lung cancer cells (A). The interaction of Ku80 and CBP was detected by IP assay using Ku80 antibody precipitated CBP from H1299, H460 and A549 cell nuclear extracts. The complex was detected by Western blot using anti-CBP antibody. IgG was used as negative control. (B). Co-localization of Ku80 and CBP in A549 and H460 cells by Immunofluorescence assay. The red staining by secondary tetra methyl rhodamine isothiocyanate was Ku80, and the green one by secondary fluorescein isothiocyanate-conjugated antibodies was CBP. (C). The nuclear extracted proteins from H460 cells treated with C464, and transfected with CBP plasmids were immunoprecipitated by anti-acetylation antibody. The complex was detected with anti-Ku80 antibody. WCL represents the whole nuclear extracts. (D). The nuclear extracted proteins from H460 cells treated with C464, and transfected with CBP plasmids were incubated with COX-2 promoter probe (−459 to +9) and streptavidin-agarose beads. The complex was detected with anti-Ku80 antibody. (E). MTT was performed in H1299 cells transfected with Ku80, Ku80 and siCBP, or Ku80 and C464. (F). The COX-2 expression in H1299 cells transfected with Ku80, Ku80 and siCBP, or Ku80 and C464 treatment after stimulation with LPS. (G). The COX-2 promoter activity was detected in H1299 cells co-transfected with COX-2 promoter (−459/+9)-driven luciferase plasmids and Ku80 plasmids, or Ku80 and siCBP or Ku80 and C646.

As a transcription co-activator, CBP has been reported to participate in the transcription of COX-2 through cooperating with NF-κB in many studies [26,27]. We therefore proposed that CBP might be involved in Ku80-mediated regulation of COX-2 expression similarly through synergy with Ku80 itself. To test this hypothesis, immunoprecipitation was performed. The nuclear extracts of lung cancer cells H1299, H460 and A549 were incubated with anti-Ku80 antibody, and then were immunoprecipitated with protein A/G-agarose beads. The complexes were then eluted and assayed by Western blot. As shown in Figure 7A, CBP protein was presented in the immune complexes precipitated by the antibodies against Ku80. Next, we further analyzed the co-localization of Ku80 with CBP in A549 and H460 cells by immunofluorescence analysis. As shown in Figure 7B, both Ku80 (red) and CBP (green) staining was detected in cell nucleus and had the same sub-cellular localization. These results indicated the existence of the interaction between Ku80 and CBP in lung cancer cells.

CBP protein is thought to increase gene expression partially through its histone acetyltransferase (HAT) activity, which could acetylate histones and relax the chromatin structure at the gene promoter. Based on the cooperation between CBP and Ku80, we further detected the acetylation of Ku80 by CBP. Compared to the control group, CBP overexpression resulted in an increase in acetylated level of Ku80, and the treatment with C646, an inhibitor of CBP HAT activity, lowered the acetylated level of Ku80 (Figure 7C). While the expression of Ku80 itself was not altered by CBP overexpression or activity inhibition by its inhibitor. These results demonstrated that CBP interacted with Ku80 and very possibly acetylated the latter to co-regulate gene expression in lung cancer cells.

To verify the role of Ku80's cooperation with CBP and the CBP-mediated acetylation of Ku80 in regulating COX-2 expression, we performed pulldown assay to analyze CBP's effect on the binding of Ku80 at COX-2 promoter region. H1299 cells were respectively transfected with LacZ or CBP plasmid or treated with CBP inhibitor. As shown in Figure 7D, the binding activity of Ku80 on COX-2 promoter in the group treated with CBP inhibitor is significant lower compared with the control cells. Similarly, its binding activity in the group transfected with CBP plasmid was highly improved than the group treated with Lac Z plasmid. The results showed that the Ku80-mediated regulation of COX-2 expression might be realized under the help of CBP's co-anchoring at COX-2 promoter region in lung cancer cells.

To further confirm the effect of CBP on the up-regulation of COX-2 expression mediated by Ku80, we detected the cell viability (Figure 7E) and COX-2 expression (Figure 7F) in H1299 cells co-transfected with Ku80 plasmids and siCBP or CBP inhibitor C646 with Ku80 plasmids. Additionally, COX-2 promoter-driven luciferase activity was also tested in lung cancer cells cotransfected with COX-2 (−459/+9 promoter region)-luciferase plasmids following the transfection with Ku80 and siCBP or C646 treatment. As shown in Figure 7E-F, cells transfected with Ku80 have higher cell viability and levels of COX-2 expression compared with cells transfected with the control plasmids. While the cells cotransfected with Ku80 and siCBP or C646 treatment showed lower cell viability and COX-2 expression level compared with the cells transfected with Ku80. Similarly, the treatment with siCBP or C646 after Ku80 transfection significantly decreased COX-2 promoter activity compared with Ku80 plasmids treatment alone (Figure 7G). All the results suggested that Ku80 promoted up-regulation of COX-2 expression through cooperation with CBP and being acetylated by the latter.

## DISCUSSION

In this study, we discovered and identified several potentially critical proteins which bound to COX-2 promoter and activated its transcription in human lung cancers using streptavidin-agarose pulldown assay and high-throughput proteomics. Ku80 was chosen because of the same trend of Ku80 and COX-2 expression in different lung normal cells and cancer cells. The expression of Ku80 is higher in human lung adenocarcinomas than that in normal lung cells, so did the expression of COX-2. Ku80 can enhance the expression of the COX-2 promoter-driven luciferase reporter gene and COX-2 protein itself, whereas the inhibition of Ku80 by its specific siRNA did the opposite. Knockdown of Ku80 also inhibited lung cancer cell growth by decreasing COX-2 expression *in vitro* and *in vivo*. Furthermore, the expression of Ku80 was positively correlated with that of COX-2 through IHC assay in lung adenocarcinoma specimens. All the results uncovered the new role of Ku80 as a tumor-specific regulator of COX-2 in lung carcinoma, and also provided a new possibility to develop Ku80 as a potential anti-cancer therapeutic target.

It is reported that the level of COX-2 expression is low in most normal tissues, while it is up-regulated in a variety of cancers and promotes their tumorigenesis and development [8, 9]. Genetic knock-out or pharmacological inhibition of COX-2 has been shown to protect against experimentally-induced carcinogenesis [47]. Furthermore, more and more studies focus on the announcement of new transcriptional regulators of COX-2 [24, 25]. Therefore, it is interesting and more significant to find out the unknown specific factors in regulating COX-2 expression in cancer cells and further explore how COX-2 is activated by these regulatory factors during tumorigenesis and development. In this study, we established the possibility of Ku80's binding to COX-2 promoter and confirmed its regulation on COX-2 expression in lung adenocarcinomas though pulldown assay, ChIP, and other series of experiments. A completely new role of Ku80 as a transcriptional factor has been revealed in our study, not only that, such transcriptional regulatory role was found to be partially realized through controlling COX-2 expression.

As a DNA repair protein, Ku80 is crucial in maintaining normal function and genetic stability of mammalian cells, acting as a tumor suppressor in carcinogenesis. However here Ku80 is reported as a tumor promoter. The contrary effects of Ku80 might be explained from two aspects. The first one is the complexity and real-time change of the cancer-related protein, just like p53, which has multiple functions in the development of cancer. At the initiation stage, Ku80 might act as a tumor suppressor in carcinogenesis and can repair the damaged DNA. With the development of tumor, the role of Ku80 in DNA damage repair might be gradually weakened, and its role in regulating the expression of the cancer-related genes such as COX-2 might be improved. The second reason might be the diversity and complexity of COX-2 transcription regulation. The expression of COX-2 is tightly controlled not only by the known protein factors such as AP-1, NF-kB, C/EBPβ and p300, but also by some unknown and new protein factors such as Ku80. These new protein factors maybe interact with the known protein factors directly or indirectly to up-regulate COX-2 expression and promote tumor growth. Further studies are still needed to explore the exact molecular mechanisms of Ku80 in the regulation of COX-2 expression and tumor growth.

CBP, a transcription co-activator, can bind to the NF-κB family proteins and co-regulate COX-2 expression. Also, it associates with SP-1 and AP-2 to co-regulate hTERT expression [26] in the previous reports. All the findings provided the possibility that CBP participated in the regulation of COX-2 through co-anchoring with Ku80 at COX-2 promoter region. We verified this hypothesis by a serial experiments, including IP and IF assay, test of the acetylated level of Ku80, and the binding assay of Ku80 at COX-2 promoter region affected by CBP level. The results show that CBP did interact with Ku80 and acetylate it to co-regulate the transcriptional activity of COX-2 in lung cancer cells.

COX-2 promoter activation and expression is regulated by multiply transcriptional factors such as AP-1, C/EBPβ and NF-κB [47, 57]. Each pro-inflammatory mediator requires binding of a combination of different transactivators to their respective enhancer elements. Several reports have also shown that transcription co-activator p300 is involved in the regulation of COX-2 expression. P300 and CBP have a high degree of sequence homology. It is generally believed that they have similar functions and play redundant roles in gene expression. Considering the complexity of the transcription regulation of COX-2, besides CBP, there must be some other protein factors involved in the association with Ku80 directly or indirectly in co-mediating COX-2 expression. Further studies will be done in our future research to explore these underlying molecular mechanisms involved in the regulation of COX-2 expression.

Our study also provided the clinical evidence that the Ku80 regulated COX-2 expression. Both Ku80 and COX-2 proteins were highly expressed in tumor tissues compared to adjacent non-malignant lung tissues. Moreover, the lung adenocarcinoma patients with high Ku80 and COX-2 expression had a significantly shorter OS than those with low Ku80 and COX-2 expression. All the results suggested the significant correlation between COX-2 and Ku80 expression (P<0.001). However, Ku80 expression shows no significant meanings to five-year survival (P>0.05) in the Cox-regression analyses for prognosis on multiple factors. It may be caused by the original function of Ku80. As one dimer of Ku, Ku80 plays a very important role in DNA damage repair, which is a crucial process in maintaining normal function and genetic stability of mammalian cells [48, 49]. Once DNA damage repair was destroyed, apoptosis might be caused or even more seriously carcinogenesis arised [50, 51]. Therefore, the lower level of Ku80 might be one reason to cause cancer. On the contrary, a new report showed that lung tumors, compared with normal tissues, exhibited a significant overexpression of a wide number of DNA damage repair genes, mostly associated with DSBR, PRR, DNA replication, and telomere maintenance pathways [36, 37]. Therefore, the expression level of Ku80 in cells should be extraordinary significant for cells' normal ability. Especially in carcinogenesis and development, it might play the role of a double-edged sword. The inconsistencies of expressions of COX-2 and Ku80 in 12 tumor samples, which contained high levels of COX-2 but low levels of Ku80, was also found in our study, suggesting that besides Ku80, there must be some other factors involved in the regulation of COX-2 expression in lung adenocarcinomas, such as NF-κB, AP-1/2, C/EBP and so on. The previous studies have revealed the diversity and complexity of COX-2 transcriptional regulation. Thus, our current study adds a new mechanism to the body of researches on the transcriptional regulation of COX-2, which is that COX-2 is a direct transcriptional target of Ku80 and its regulation is partially and might be critically controlled by Ku80 in lung adenocarcinoma cells.

In summary, our study verifies that Ku80 up-regulates COX-2 promoter activity and further activates the expression of COX-2 in lung cancer cells. Such transcriptional activation ability of Ku80 in mediating COX-2 gene expression may be realized through co-auchoring with CBP at COX-2 promoter elements and being acetylated by the latter. Ku80 knockdown inhibited lung tumor growth *in vitro* and *in vivo*. The lung adenocarcinoma patients with high expression levels of Ku80 and COX-2 protein had shorter survival periods. All the evidence provided the potential to develop Ku80 as a useful therapeutic target in some key stages of lung cancer progression.

## MATERIALS AND METHODS

### Cell lines and cell culture

Human lung cancer cell lines (H1299, A549, H322, H460) were obtained from the American Type Culture Collection (ATCC, Manassas, VA) and cultured in RPMI-1640 medium (Gibco), supplemented with 10% fetal bovine serum. Normal human bronchial epithelial cell line (HBE) and human lung fibroblast cell (HLF) were also got from ATCC and maintained in Dulbecco's modified Eagle's medium supplemented with 10% fetal bovine serum. All the cells were incubated at 37°C in a humidified atmosphere with 5% carbon dioxide

### Streptavidin-agarose pulldown assay

Streptavidin-agarose pulldown assays were done as described previously [47]. The biotin-labeled double-stranded oligonucleotide probe, which corresponds to −30/-508 fragments of COX-2 promoter sequence were synthesized by TAKARA Company (sense, 5′-ACGTGACTTCCTCGACCCTC-3′; antisense, 5′-AAGACTGAAAACCAAGCCCA-3′). The assay was performed by mixing 400 μg nuclear proteins from different cell lines, 4 μg of the double-strand biotinylated probe and 50 μl of steptavidin-agarose beads solution (Sigma) and was incubated on a rotating wheel at room temperature for 2 hours. The beads were then pelleted by centrifugation at 600×g for 1 min and then washed 3 times with 200 μl PBSI each time. The collected beads were finally resuspended with 30 μl loading buffer and cooked at 100 °C for 5 min. The supernatant containing the bound proteins was separated by SDS-PAGE for further analysis.

### Identification of COX-2 promoter-binding proteins

The COX-2 promoter-binding proteins were separated by 10% SDS-PAGE and visualized by silver staining according to the suggested protocol (Beyotime, China). The protein bands of interest in the gel were cut and digested with trypsin. The identification of digested samples was performed through mass spectrometry analysis. The proteins indicated by the bands of interest were further identified via searching the available proteomics databases.

### Western blot analysis

Western blot analyses were performed according to the protocols for the routine with antibodies against Ku80, COX-2 (Abcam), CBP (CST, USA), β-actin and GAPDH (Proteintech group, CA) respectively.

### Chromatin immunoprecipitation assay (ChIP)

The ChIP assay was performed as previously described [52]. Briefly, 1% formaldehyde was added to the culture medium of cells for 10mins, and 0.125M glycine was added to stop cross-linking. The cells were rinsed twice with ice-cold PBS, scraped, and collected by centrifugation. The cells were resuspended with 500 μl IP Buffer (100 mM NaCl, 5 mM EDTA, 10 mM Tris-HCl, pH 8.0, 0.02% NaN_3_, 10% SDS, 5.0% Triton X-100) for sonication for three times with 20s each, and the debris was removed by centrifugation. 50 μl lysate was used as the DNA input control and the remaining was incubated with anti-Ku80 antibody or non-immune rabbit IgG overnight at 4°C. Immunoprecipitated complexes were collected using protein A/G agarose beads and were extensively washed with Micelle Wash Buffer, Buffer 500, LiCl/detergent solution, TE Buffer, respectively. Then 1% SDS and 0.1 M NaHCO_3_ was added to the immunoprecipitates to be incubated at 65°C overnight, followed by treatment with 400 μg/ml proteinase K for 2 h at 37°C. The DNA was extracted with phenol/chloroform and precipitated with ethanol. Final pellets were resuspended in 100 μl ddH_2_O and subjected to PCR amplification using specific COX-2 promoter primers (5′-primer, ACGTGACTTCCTCGACCCT C; and 3′-primer, CAGGCGCACAGGTTTCCGCC). The resulting product of 238 bp for the COX-2 promoter was separated by 1% agarose gel electrophoresis.

### Plasmid vector

A 5′-flanking DNA fragment from position −891 to +9 (−891/+9, −459/+9, −363/+9, −193/+9, −96/+9, −53/+9) of human COX-2 gene was constructed into a promoter luciferase expression vector, pGL3 [44, 52]. The Ku80 and CBP expression vectors pEGFP-C1-FLAG-Ku80 and FLAG-CBP and the control vector FLAG-lacZ were designed and purchased from Addgene (US).

### siRNA design and transfection

The siRNAs targeting Ku80 (siRNA1: 5′-GGCUCCAAUUUGUCUAUAATT-3′; 5′-UUAUAGACAAAUUGGAGCCTT-3′. siRNA2: 5′-GGUGGCCAUAGUUCGAUAUTT-3′; 5′-AUAUCGAACUAUGGCCACCTT-3′. siRNA3: 5′ GAGCAGCGCUUUAACAACUTT-3′; 5′-AGUUGUUAAAGCGCUGCUCTT-3′.), siRNA targeting CBP (5′-GAGGUCGUUUACAUAAATT-3′; 5′-UUUAUGUAAACGCGACCUCTT-3′) and negative control siRNA(5′-UUCUCCGAACGUGUCACGUTT-3′;5′-ACGUGACACGUUCGGAGAATT-3′) were purchased from ShangHai GenePharma Co (Shanghai, China). Cells plated in 96-well plates (5,000 cells/well) or six-well plates (200,000 cells/well) were transfected with siRNA duplexes (1-2 μg) encapsulated by DC-nanoparticles. At 48 hours after treatment, protein expression and cell viability were tested by Western blot, RT-PCR and MTT analysis, respectively.

### Nuclear extraction

Nuclear extraction was performed as previously described [53]. Cells were lysed in 250 μl cytoplasm lysis buffer (10 mM Hepes, pH 7.9, 10 mM KCl, 1.5 mM MgCl_2_·6H_2_O, 0.5% NP-40, 300mM Sucrose) with multiple protease inhibitors(1 mM Na_3_VO_4_, 10 mM NaF, 2.5 mM β-glycerophosphate, 0.1 mM PMSF, 1 g/ml leupeptin, and 0.5 mM dithiothreitol) on ice for 10 min. The mixture was vortexed briefly, and centrifuged at 2600×g for 1 min at 4 °C. The supernatant was removed to a new tube, and stored at −80 °C. The pellet was resuspended with 70-100 ul Nuclei lysis buffer (20 mM Hepes, pH 7.9, 420 mM NaCl, 1.5 mM MgCl_2_·6H_2_O, 0.1 mM EDTA, 2.5% Glycerol) with multiple protease inhibitors and kept on ice for 30 mins. Nuclei proteins were extracted by centrifuge at 10400×g for 10 min at 4 °C. The supernatant was nuclei extracts. Protein concentration was determined by BCA assay.

### Analysis of COX-2 promoter activity

Cells (200,000 cells/well) plated in six-well plates were transfected with the COX-2 promoter luciferase plasmids encapsulated with DC-nanoparticles. Meanwhile, cells were co-transfected with either Ku80 overexpression vector (pEGFP-C1-FLAG-Ku80) or with lacZ overexpression vector used as control (FLAG-lacZ) or Ku80-specific siRNA or negative control siRNA used as control. 48 hours after treatment, the expressed luciferase activity was measured as described using a DUAL-luciferase reporter assay kit [54] (BioVision, Inc.CA, USA). The ratio of firefly luciferase to Renilla luciferase activity (relative luciferase activity) was calculated to correct the variations in transfection efficiency.

### RT-PCR

Total RNA was prepared from cultured cell lines by using Trizol Reagent (TaKaRa Bio.) according to the manufacturer's instructions. The PCR primers corresponding to COX-2, Ku80 and GAPDH functional gene sequences were synthesized by TaKaRa, and the sequences were as following: for COX-2 (sense:5′-TCACAGGCTTCCATTGACCAG-3′,antisense:5′-CCGAGGCTTTTCTACCAGA-3′, for Ku80 (sense:5′-TGACTTCCTGGATGCACTAATCGT-3′; 5′-TTGGAGCCAATGGTCAGTCG-3′), for GAPDH (sense:5′–AATCCCATCACCATCTTCC-3′;antisense:5′-CATCACGCCACAGTTTCC-3′). RT-PCR was carried out as described before [55]. The samples were first denatured at 98°Cfor 3 min, followed by 30 PCR cycles, each with temperature variations as follows: 98°C for 10 s, 58°C or other annealing temperature for 30 s, and 72°C for 30 s. The PCR products were visualized under ultraviolet light and the band density was measured through quantitative analysis.

### Immunofluorescence and confocal microscopy

The Cells were seeded onto coverslips in a 6-well plate and fixed with 4% paraformaldehyde (w/v) for 30 mins, and then were washed for 10 mins with PBS and permeabilized with 0.2% (w/v) Triton X-100 in PBS for 5 mins. The blocking step was performed for 30 min in PBS containing 1% bovine serum albumin (BSA). Cells were then incubated overnight with the primary Ku80 or CBP antibodies diluted in PBS containing 10% BSA. After being washed with PBS, cells were incubated for 1 h with secondary fluorescein isothiocyanate or tetra methyl rhodamine isothiocyanate-conjugated antibodies. After several additional washing steps, the cells were stained with DAPI (Beyotime, China). The localization of Ku80 and CBP protein was assessed using a Leica DM 14000B confocal microscopy.

### MTT assay for cell proliferation

Cell viability was determined by the MTT assay (Roche Diagnosis, Indianapolis, IN). Briefly, the H1299 and H460 cell lines seeded in 96-well plates (2,000 cells/well) were treated with siRNA of Ku80 or negative control siRNA at the indicated doses. 48 hours after treatment, the cell viability was determined.

### Clone formation assay

To analyze the effect of Ku80 on the clonogenicity of tumor cells *in vitro*, we transfected H460 cells (1,000 cells/well) seeded in six-well plates with siRNA of Ku80 or control siRNA using DC nanoparticles. After 14 days, the cells were washed with PBS and fixed with fixation solution (methanol: glacial: acetic 1:1:8) for 10 min, and stained with 0.1% crustal violet for 30 mins. The clones with more than 50 cells were counted under an optical microscope.

### *In vitro* migration assay

Scratch assay (wound healing assay) was performed to detect cell migration. The cells were grown to full confluence in six-well plates and wounded with a sterile 100 μL pipette tip after 4 h of serum starvation and then transfected with 1 μg/mL siKu80 for 8 h. Then refresh with full medium and keep in a CO_2_ incubator. After 48 h, medium was replaced with phosphate buffered saline (PBS) buffer, the wound gap was observed, and cells were photographed using a Leica DM 14000B microscope fitted with digital camera.

### Tissue microarray and immunohistochemistry analysis

The human lung adenocarcinoma tissue microarray used for immunostaining analysis of Ku80 and COX-2 protein expression was purchased from Shanghai Outdo Biotech (Shanghai, China) and contains 72 lung adenocarcinomas and their corresponding adjacent non-malignant lung tissues. The overall survival (OS) for the corresponding patients was calculated from the day of surgery to the day of death or to the last follow-up. The tissue microarray (TMA) slides were deparafﬁnized in xylene, rehydrated in graded alcohol, submerged into EDTA antigenic retrieval buffer and microwaved for antigenic retrieval, followed by treatment with 3% hydrogen peroxide in methanol to quench the endogenous peroxidase activity and incubation with 3% bovine serum albumin to block the nonspecific binding. Rabbit polyclonal anti-Ku80 (1:100; Santa Cruz) and COX-2 (1:200; Abcam) antibody were incubated with the TMA overnight at 4 °C. For negative controls, the primary antibody was replaced by normal rabbit serum and then were treated with biotinylated anti-rabbit secondary antibody (protein tech, US), followed by incubation with streptavidin horseradish peroxidase complex (CST). The degrees of immunostaining were reviewed and scored by two independent observers. The proportion of the stained cells and the extent of the staining were used as criteria of evaluation. For each case, at least 1,000 tumor cells were analyzed and the percentage of the tumor cells with positively stained nuclear was recorded. For each sample, the proportion of Ku80 and COX-2-expressing cells varied from 0% to 100%, and the intensity of nuclear staining varied from weak to strong. One score was given according to the percentage of positive cells as:<5% of the cells:1 point; 6-35% of the cells:2 point; 36-70% of the cells:3 point; >70% of the cells: 4 point. Another score was given according to the intensity of staining as: negative staining: 1 point; weak staining (light yellow): 2 point; moderate staining (yellowish brown): 3 point; and strong staining (brown): 4 point. A final score was then calculated by multiple the above two scores. If the final score was equal or bigger than four, the protein expression in the tumor was considered high; otherwise, the protein expression in the tumor was considered low [56].

### Acquisition of carcinoma tissue samples

Lung cancer samples and adjacent non-carcinoma tissues were collected at the first affiliated hospital of Dalian Medical University (Dalian, China) from patients of squamous cell carcinoma with different histological types (n=3). All the samples were stored at −80°C until western blot analysis. Informed consent was obtained from each patient and the whole study was approved by the Committees on Human Rights in Research at Dalian Medical University.

### Xenograft mouse model and tumor/tissue processing

Animal experiments were carried out in accordance with the National Institute of Health Guide for the Care and Use of Laboratory Animals under the approval of the SPF Laboratory Animal Center at Dalian Medical University [57]. H460 cells (5×10^6^) were inoculated subcutaneously into the armpit of the nude mice. Once palpable tumors were observed, tumor volume measurements were taken every four days using calipers. Mice were randomly divided into 4 groups (5 mice per group) after the tumor volume reached 50 mm^3^,: (a) control siRNA; (b) Ku80 siRNA; (c) Ku80 siRNA+ LPS(10 μg / kg body weight [58]); (d) control siRNA + LPS. For delivery of DC nanoparticles-conjugated siRNA, 10 μg siRNA in 0.1 ml saline buffer was injected intratumorally twice a week for 3 weeks [59]. The tumor volume was calculated as V= (width^2^×length)/2 using digital calipers. At last, the mice were sacrificed and the tumor size and weight was photographed and recorded respectively.

### Detection of Ku80 and COX-2 expression in xenograft tumor tissues

Tumor tissues from the above treated animals were collected and placed in 10% formalin and were further embedded in paraffin for the following analysis. One part were used for protein extraction and further analyzed by western blot. The rest were embedded for histological analysis. A negative control was obtained by replacing the primary antibody of Ku80 or COX-2 with a normal rabbit or mouse IgG. The positive immunoreactive cells from each of the differently treated tumor tissue sections were measured at 200x magnification using a light microscope. The amount of proteins was analyzed by integral optical density (IOD) using IPP software (Image Plus Pro 6.0, Bethesda, MD, USA).

### Co-Immunoprecipitation

To determine the interaction of CBP with Ku80, immunoprecipitation was performed as previously described. Nuclear extract proteins (300μg) prepared from H1299 cells were incubated respectively with a specific rabbit polyclonal antibody to Ku80 or a nonimmune rabbit IgG at a final concentration of 1 g/mL each overnight at 4°C. The immune complexes were pulled down by protein A/G agarose beads (Santa Cruz Biotechnology) and washed with PBS buffer containing proteinase inhibitor 3 times. The immunoprecipitated proteins were separated by SDS-PAGE and analyzed by Western blotting using a CBP antibody.

### Determination of acetylated Ku80

The nuclear extracts were immunoprecipitated with a specific antibody of pan-Acety or a nonimmune IgG as control and the immunoprecipitates were pulled down with protein A/G agarose beads. After extensive washing, the precipitated proteins were separated by SDS-PAGE gel and analyzed by Western blotting using a Ku80 antibody.

### Statistical analysis

Student's t-tests were used to compare two independent groups of data. Survival curves were constructed using the Kaplane Meier method and were compared using the log-rank test. Statistical analyses were performed using the SPSS 16.0 software. The results were reported as the mean SE±SD. Values of P < 0.05 were considered to be statistically significant.
